# Bis[μ-1,2-bis­(diphenyl­phosphino)ethane-κ^2^ *P*:*P*′]digold(I)(*Au*—*Au*) bis­(trifluoro­methane­sulfonate) acetonitrile disolvate

**DOI:** 10.1107/S1600536809025513

**Published:** 2009-07-11

**Authors:** Christoph E. Strasser, Stephanie Cronje, Helgard G. Raubenheimer

**Affiliations:** a Department of Chemistry and Polymer Science, University of Stellenbosch, Private Bag X1, Matieland, 7602, South Africa

## Abstract

The title compound, [Au_2_(C_26_H_24_P_2_)_2_](CF_3_SO_3_)_2_·2CH_3_CN, comprises a cyclic cation with a short intra­molecular aurophilic inter­action of 2.9220 (3) Å. The trifluoro­methane­sulfonate anions and acetonitrile solvent mol­ecules are located in channels formed by the complex cations that run along the crystallographic *c* axis. Each counter-anion is also engaged in a C—H⋯O contact with one of the methyl­ene H atoms of a 1,2-bis­(diphenyl­phosphino)ethane (dppe) ligand; another C—H⋯O contact involving an aromatic H atom is also observed.

## Related literature

For ^31^P NMR evidence of [Au_2_(μ-dppe)_3_]^2+^, see: Al-Baker *et al.* (1985 ▶). For [Au_2_(μ-dppm)_2_]^2+^, see: de Jongh *et al.* (2007 ▶). For a related structure, see: Schuh *et al.* (2001 ▶).
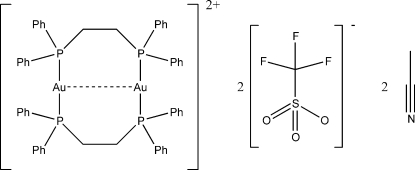


## Experimental

### 

#### Crystal data

[Au_2_(C_26_H_24_P_2_)_2_](CF_3_SO_3_)_2_·2C_2_H_3_N
*M* *_r_* = 1571.0
Monoclinic, 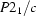

*a* = 11.7888 (9) Å
*b* = 36.998 (3) Å
*c* = 14.377 (1) Å
β = 113.011 (1)°
*V* = 5771.6 (8) Å^3^
*Z* = 4
Mo *K*α radiation
μ = 5.33 mm^−1^
*T* = 100 K
0.21 × 0.15 × 0.07 mm

#### Data collection

Bruker SMART APEX CCD area-detector diffractometer
Absorption correction: multi-scan (*SADABS*; Bruker, 2002 ▶) *T*_min_ = 0.404, *T*_max_ = 0.686
36089 measured reflections
13385 independent reflections
10742 reflections with *I* > 2σ(*I*)
*R*_int_ = 0.048

#### Refinement

*R*[*F* ^2^ > 2σ(*F* ^2^)] = 0.042
*wR*(*F* ^2^) = 0.095
*S* = 1.01
13385 reflections
723 parameters
H-atom parameters constrained
Δρ_max_ = 2.35 e Å^−3^
Δρ_min_ = −0.75 e Å^−3^

### 

Data collection: *SMART* (Bruker, 2002 ▶); cell refinement: *SAINT* (Bruker, 2003 ▶); data reduction: *SAINT*; program(s) used to solve structure: *SHELXS97* (Sheldrick, 2008 ▶); program(s) used to refine structure: *SHELXL97* (Sheldrick, 2008 ▶); molecular graphics: *X-SEED* (Barbour, 2001 ▶; Atwood & Barbour, 2003 ▶); software used to prepare material for publication: *X-SEED*.

## Supplementary Material

Crystal structure: contains datablocks I, global. DOI: 10.1107/S1600536809025513/im2111sup1.cif

Structure factors: contains datablocks I. DOI: 10.1107/S1600536809025513/im2111Isup2.hkl

Additional supplementary materials:  crystallographic information; 3D view; checkCIF report

## Figures and Tables

**Table 1:** Hydrogen-bond geometry (Å, °)

*D*—H⋯*A*	*D*—H	H⋯*A*	*D*⋯*A*	*D*—H⋯*A*
C212—H212⋯O1^i^	0.95	2.45	3.387 (7)	171
C21—H21*B*⋯O1^i^	0.99	2.34	3.268 (7)	155
C11—H11*B*⋯O4^ii^	0.99	2.36	3.301 (7)	158

Symmetry codes: (i) 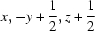; (ii) 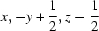.

**Fig. 1.**
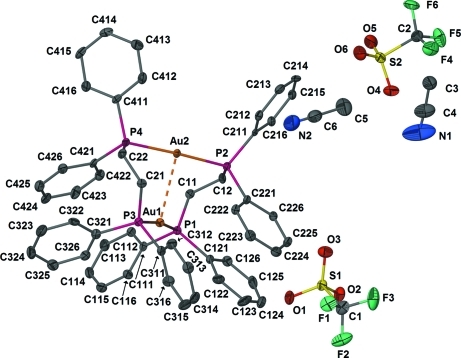
The asymmetric unit of (I), ellipsoids are drawn at the 50% probability level.

## supplementary crystallographic information

### Comment

Ditopic phosphines can form cyclic cations with gold(I) and especially dppm [bis(diphenylphosphino)methane] readily yields the [Au_2_(µ-dppm)_2_]^2+^ cation (de Jongh *et al.*, 2007; and references cited therein). However, the tendency of dppe [1,2-bis(diphenylphosphino)ethane] to form cyclic cations is less apparent than that of dppm since only one structural report of a bis(methanol) solvate, [Au_2_(µ-dppe)_2_](CF_3_SO_3_)_2_.CH_3_OH, has been published (Schuh *et al.*, 2001). The two solvates, however are not isomorphous with (I) being monoclinic and the methanol solvate triclinic.

Compound (I) crystallizes as an asymmetric cation with the trifluoromethanesulfonate anions forming two sets of channels running parallel to the crystallographic *a* axis (for the anion containing S1) and *c* axis (for the anion containing S2), respectively. The acetonitrile containing N2 is also found in the former channels while another solvent is embedded between the cations.

Compared to the other example of a crystallographically characterized [Au_2_(µ-dppe)_2_]^2+^ cation in literature, (I) (Figure 1) exhibits a shorter aurophilic interaction and slightly wider P1—Au1···Au2—P2 and P3—Au1···Au2—P4 torsion angles [2.9220 (3) Å, -47.11 (5) and -46.89 (5)° in (I) compared to 2.959 (1) Å, -43.8 (1) and -45.0 (1)°, in the example of Schuh *et al.*]. The angles at the gold centres in (I) are bent significantly from the linear ideal [P1—Au1—P3 171.77 (5)° and P2—Au2—P4 177.10 (5)°] due to the attractive aurophilic interaction. Other geometric parameters between both structures agree very closely and differences would likely be caused by lattice effects. Another noteworthy feature of (I) is the well defined trifluoromethanesulfonate anions and acetonitrile solvent molecules that do not exhibit disorder despite the fact that the thermal displacement ellipsoids of the acetonitrile containing N1 show higher mobility. Disorder of one trifluoromethanesulfonate anion and methanol molecule each was observed in the crystal structure of the bis(methanol) solvate which may have been enhanced by the higher temperature [223 (2) K] at which data were collected.

The title compound (I) was obtained as the exclusive product in an unsuccessful attempt to structurally characterize the [Au_2_(µ-dppe)_3_]^2+^ cation that has been previously detected by ^31^P NMR spectroscopy (Al-Baker *et al.*, 1985) and its presence in the mother liquor of (I) can therefore not be completely ruled out.

### Experimental

The ditopic phosphine dppe (184 mg, 0.46 mmol) was suspended in 20 ml of acetonitrile, sodium trifluoromethanesulfonate (53 mg, 0.31 mmol) was added and the suspension stirred briefly. [AuCl(tht)] (99 mg, 0.31 mmol; tht = tetrahydrothiophene) and few NaCl crystals (to seed precipitation) were subsequently added. After 1 h the precipitated solids were filtered off, the filtrate was reduced to *ca* 5 ml and layered with diethyl ether. Colourless blocks of (I) crystallized at 258 K. No other species could be identified in the crystalline phase.

### Refinement

All H atoms were positioned geometrically (C—H = 0.95, 0.99 and 0.98 Å for CH, CH_2_ and CH_3_ groups, respectively) and constrained to ride on their parent atoms; *U*_iso_(H) values were set at 1.2 times *U*_eq_(C) for CH and CH_2_ groups and 1.5 times *U*_eq_(C) for CH_3_ groups.

The maximum residual electron density of 2.35 e Å^-3^ is located 0.85 Å next to Au1.

### Crystal data

**Table:**

[Au_2_(C_26_H_24_P_2_)_2_](CF_3_SO_3_)_2_·2C_2_H_3_N	*F*(000) = 3072
*M**_r_* = 1571.0	*D*_x_ = 1.808 Mg m^−^^3^
Monoclinic, *P*2_1_/*c*	Melting point: 528 K
Hall symbol: -P 2ybc	Mo *K*α radiation, λ = 0.71073 Å
*a* = 11.7888 (9) Å	Cell parameters from 6846 reflections
*b* = 36.998 (3) Å	θ = 2.2–27.1°
*c* = 14.377 (1) Å	µ = 5.33 mm^−^^1^
β = 113.011 (1)°	*T* = 100 K
*V* = 5771.6 (8) Å^3^	Block, colourless
*Z* = 4	0.21 × 0.15 × 0.07 mm

### Data collection

**Table:**

Bruker SMART APEX CCD area-detector diffractometer	13385 independent reflections
Radiation source: fine-focus sealed tube	10742 reflections with *I* > 2σ(*I*)
graphite	*R*_int_ = 0.048
ω scans	θ_max_ = 28.2°, θ_min_ = 1.6°
Absorption correction: multi-scan (*SADABS*; Bruker, 2002)	*h* = −15→14
*T*_min_ = 0.404, *T*_max_ = 0.686	*k* = −49→36
36089 measured reflections	*l* = −19→19

### Refinement

**Table:**

Refinement on *F*^2^	Primary atom site location: structure-invariant direct methods
Least-squares matrix: full	Secondary atom site location: difference Fourier map
*R*[*F*^2^ > 2σ(*F*^2^)] = 0.042	Hydrogen site location: inferred from neighbouring sites
*wR*(*F*^2^) = 0.095	H-atom parameters constrained
*S* = 1.01	*w* = 1/[σ^2^(*F*_o_^2^) + (0.0437*P*)^2^] where *P* = (*F*_o_^2^ + 2*F*_c_^2^)/3
13385 reflections	(Δ/σ)_max_ = 0.002
723 parameters	Δρ_max_ = 2.35 e Å^−^^3^
0 restraints	Δρ_min_ = −0.75 e Å^−^^3^

### Special details

**Table:**

Geometry. All e.s.d.'s (except the e.s.d. in the dihedral angle between two l.s. planes) are estimated using the full covariance matrix. The cell e.s.d.'s are taken into account individually in the estimation of e.s.d.'s in distances, angles and torsion angles; correlations between e.s.d.'s in cell parameters are only used when they are defined by crystal symmetry. An approximate (isotropic) treatment of cell e.s.d.'s is used for estimating e.s.d.'s involving l.s. planes.
Refinement. Refinement of *F*^2^ against ALL reflections. The weighted *R*-factor *wR* and goodness of fit *S* are based on *F*^2^, conventional *R*-factors *R* are based on *F*, with *F* set to zero for negative *F*^2^. The threshold expression of *F*^2^ > 2σ(*F*^2^) is used only for calculating *R*-factors(gt) *etc*. and is not relevant to the choice of reflections for refinement. *R*-factors based on *F*^2^ are statistically about twice as large as those based on *F*, and *R*- factors based on ALL data will be even larger.

### Fractional atomic coordinates and isotropic or equivalent isotropic displacement parameters (Å^2^)

**Table:**

	*x*	*y*	*z*	*U*_iso_*/*U*_eq_	
Au1	0.623479 (18)	0.134499 (5)	0.336490 (15)	0.01447 (6)	
S1	0.51439 (13)	0.41844 (4)	0.19532 (10)	0.0215 (3)	
P1	0.54886 (12)	0.18698 (4)	0.24655 (10)	0.0140 (3)	
F1	0.7019 (3)	0.38763 (10)	0.3408 (3)	0.0412 (9)	
O1	0.5380 (4)	0.39408 (11)	0.1268 (3)	0.0321 (10)	
N1	0.0693 (7)	0.4616 (3)	0.4339 (7)	0.111 (4)	
C1	0.6633 (6)	0.42044 (17)	0.3017 (5)	0.0338 (15)	
Au2	0.415549 (18)	0.128908 (5)	0.398184 (15)	0.01447 (6)	
S2	0.05080 (14)	0.32922 (4)	0.64951 (11)	0.0270 (3)	
P2	0.43840 (12)	0.18914 (4)	0.44274 (10)	0.0137 (3)	
F2	0.7494 (4)	0.43370 (12)	0.2719 (3)	0.0576 (12)	
O2	0.4945 (4)	0.45535 (11)	0.1614 (3)	0.0310 (10)	
N2	0.1128 (5)	0.3048 (2)	0.3405 (5)	0.0543 (18)	
C2	0.0292 (5)	0.34533 (17)	0.7605 (5)	0.0288 (14)	
P3	0.71663 (12)	0.08150 (4)	0.41409 (10)	0.0147 (3)	
F3	0.6598 (4)	0.44111 (12)	0.3755 (3)	0.0591 (13)	
O3	0.4330 (4)	0.40469 (12)	0.2393 (3)	0.0341 (10)	
C3	−0.0048 (7)	0.4312 (2)	0.5593 (6)	0.0466 (18)	
H3A	−0.0708	0.4143	0.5219	0.070*	
H3B	−0.0361	0.4491	0.5937	0.070*	
H3C	0.0639	0.4179	0.6094	0.070*	
P4	0.39442 (12)	0.06958 (4)	0.34652 (10)	0.0150 (3)	
F4	0.1308 (3)	0.34219 (12)	0.8437 (3)	0.0470 (11)	
O4	0.1513 (4)	0.35096 (12)	0.6475 (3)	0.0370 (11)	
C4	0.0371 (7)	0.4493 (2)	0.4906 (6)	0.055 (2)	
F5	−0.0018 (4)	0.38046 (11)	0.7517 (3)	0.0530 (12)	
O5	−0.0660 (4)	0.33678 (14)	0.5692 (3)	0.0416 (12)	
C5	0.0301 (8)	0.3640 (2)	0.3840 (6)	0.058 (2)	
H5A	0.0973	0.3815	0.4115	0.088*	
H5B	−0.0045	0.3589	0.4344	0.088*	
H5C	−0.0343	0.3739	0.3229	0.088*	
F6	−0.0600 (3)	0.32773 (11)	0.7752 (3)	0.0432 (10)	
O6	0.0822 (4)	0.29207 (12)	0.6734 (4)	0.0435 (12)	
C6	0.0770 (7)	0.3311 (2)	0.3592 (5)	0.0416 (17)	
C11	0.3965 (4)	0.20169 (14)	0.2369 (4)	0.0154 (11)	
H11A	0.3659	0.2199	0.1823	0.018*	
H11B	0.3404	0.1806	0.2152	0.018*	
C12	0.3855 (5)	0.21770 (14)	0.3307 (4)	0.0172 (11)	
H12A	0.2979	0.2238	0.3138	0.021*	
H12B	0.4328	0.2406	0.3476	0.021*	
C21	0.6443 (5)	0.05786 (14)	0.4879 (4)	0.0160 (11)	
H21A	0.7009	0.0383	0.5256	0.019*	
H21B	0.6387	0.0751	0.5386	0.019*	
C22	0.5162 (5)	0.04105 (13)	0.4334 (4)	0.0176 (11)	
H22A	0.4879	0.0320	0.4856	0.021*	
H22B	0.5253	0.0198	0.3951	0.021*	
C111	0.5323 (5)	0.17899 (13)	0.1181 (4)	0.0146 (10)	
C112	0.4219 (5)	0.18370 (14)	0.0346 (4)	0.0170 (11)	
H112	0.3510	0.1921	0.0439	0.020*	
C113	0.4150 (5)	0.17621 (15)	−0.0620 (4)	0.0231 (12)	
H113	0.3400	0.1800	−0.1188	0.028*	
C114	0.5184 (5)	0.16318 (15)	−0.0755 (4)	0.0237 (12)	
H114	0.5130	0.1571	−0.1413	0.028*	
C115	0.6284 (5)	0.15913 (15)	0.0063 (4)	0.0240 (12)	
H115	0.6997	0.1513	−0.0033	0.029*	
C116	0.6350 (5)	0.16644 (14)	0.1019 (4)	0.0203 (12)	
H116	0.7107	0.1629	0.1581	0.024*	
C121	0.6486 (5)	0.22545 (14)	0.2910 (4)	0.0157 (11)	
C122	0.7689 (5)	0.22112 (17)	0.3589 (4)	0.0247 (13)	
H122	0.8006	0.1977	0.3814	0.030*	
C123	0.8436 (6)	0.25158 (19)	0.3942 (5)	0.0362 (16)	
H123	0.9263	0.2488	0.4411	0.043*	
C124	0.7982 (6)	0.28533 (18)	0.3617 (5)	0.0340 (16)	
H124	0.8496	0.3059	0.3865	0.041*	
C125	0.6787 (6)	0.28986 (16)	0.2932 (5)	0.0299 (14)	
H125	0.6481	0.3134	0.2707	0.036*	
C126	0.6040 (5)	0.26026 (15)	0.2576 (4)	0.0211 (12)	
H126	0.5217	0.2634	0.2101	0.025*	
C211	0.3496 (4)	0.20220 (13)	0.5156 (4)	0.0132 (10)	
C212	0.3608 (5)	0.18048 (14)	0.5987 (4)	0.0175 (11)	
H212	0.4141	0.1601	0.6150	0.021*	
C213	0.2948 (5)	0.18857 (16)	0.6568 (4)	0.0235 (13)	
H213	0.3047	0.1742	0.7143	0.028*	
C214	0.2145 (5)	0.21744 (16)	0.6315 (4)	0.0237 (13)	
H214	0.1678	0.2226	0.6709	0.028*	
C215	0.2014 (5)	0.23887 (15)	0.5494 (4)	0.0231 (12)	
H215	0.1468	0.2589	0.5332	0.028*	
C216	0.2677 (5)	0.23123 (14)	0.4904 (4)	0.0176 (11)	
H216	0.2573	0.2457	0.4331	0.021*	
C221	0.5969 (5)	0.20160 (13)	0.5168 (4)	0.0147 (11)	
C222	0.6842 (5)	0.17419 (14)	0.5606 (4)	0.0178 (11)	
H222	0.6596	0.1496	0.5521	0.021*	
C223	0.8067 (5)	0.18318 (16)	0.6163 (4)	0.0224 (12)	
H223	0.8659	0.1647	0.6456	0.027*	
C224	0.8423 (5)	0.21888 (16)	0.6291 (4)	0.0233 (12)	
H224	0.9261	0.2249	0.6670	0.028*	
C225	0.7564 (5)	0.24615 (15)	0.5867 (4)	0.0224 (12)	
H225	0.7816	0.2707	0.5958	0.027*	
C226	0.6345 (5)	0.23751 (13)	0.5316 (4)	0.0166 (11)	
H226	0.5759	0.2562	0.5036	0.020*	
C311	0.8709 (5)	0.09196 (14)	0.5052 (4)	0.0171 (11)	
C312	0.9202 (5)	0.07739 (15)	0.6013 (4)	0.0237 (12)	
H312	0.8720	0.0616	0.6232	0.028*	
C313	1.0394 (5)	0.08574 (16)	0.6656 (4)	0.0246 (13)	
H313	1.0726	0.0753	0.7312	0.029*	
C314	1.1105 (5)	0.10878 (16)	0.6365 (4)	0.0281 (14)	
H314	1.1926	0.1142	0.6811	0.034*	
C315	1.0600 (6)	0.12436 (16)	0.5395 (5)	0.0314 (15)	
H315	1.1077	0.1406	0.5183	0.038*	
C316	0.9410 (5)	0.11595 (15)	0.4754 (4)	0.0236 (12)	
H316	0.9066	0.1267	0.4103	0.028*	
C321	0.7306 (5)	0.04977 (14)	0.3231 (4)	0.0184 (11)	
C322	0.7267 (5)	0.01260 (15)	0.3364 (4)	0.0206 (12)	
H322	0.7189	0.0034	0.3953	0.025*	
C323	0.7340 (5)	−0.01093 (16)	0.2645 (5)	0.0310 (15)	
H323	0.7294	−0.0363	0.2729	0.037*	
C324	0.7480 (5)	0.00253 (18)	0.1808 (5)	0.0328 (15)	
H324	0.7550	−0.0137	0.1320	0.039*	
C325	0.7521 (5)	0.03941 (17)	0.1665 (4)	0.0294 (14)	
H325	0.7619	0.0484	0.1082	0.035*	
C326	0.7420 (5)	0.06317 (15)	0.2373 (4)	0.0227 (12)	
H326	0.7429	0.0885	0.2270	0.027*	
C411	0.2566 (5)	0.04865 (14)	0.3496 (4)	0.0169 (11)	
C412	0.2230 (5)	0.05834 (15)	0.4281 (4)	0.0231 (12)	
H412	0.2696	0.0760	0.4757	0.028*	
C413	0.1218 (5)	0.04255 (16)	0.4376 (5)	0.0269 (13)	
H413	0.0996	0.0490	0.4922	0.032*	
C414	0.0529 (5)	0.01721 (16)	0.3673 (4)	0.0265 (13)	
H414	−0.0174	0.0066	0.3731	0.032*	
C415	0.0858 (5)	0.00731 (16)	0.2890 (4)	0.0266 (13)	
H415	0.0388	−0.0103	0.2413	0.032*	
C416	0.1883 (5)	0.02315 (15)	0.2798 (4)	0.0236 (12)	
H416	0.2112	0.0164	0.2258	0.028*	
C421	0.3955 (4)	0.06367 (13)	0.2221 (4)	0.0151 (11)	
C422	0.3721 (5)	0.09313 (15)	0.1579 (4)	0.0216 (12)	
H422	0.3519	0.1158	0.1782	0.026*	
C423	0.3780 (5)	0.08951 (16)	0.0631 (4)	0.0282 (14)	
H423	0.3601	0.1096	0.0186	0.034*	
C424	0.4097 (5)	0.05691 (16)	0.0344 (4)	0.0277 (14)	
H424	0.4172	0.0548	−0.0288	0.033*	
C425	0.4304 (5)	0.02744 (16)	0.0964 (4)	0.0264 (13)	
H425	0.4491	0.0048	0.0746	0.032*	
C426	0.4246 (5)	0.03023 (14)	0.1899 (4)	0.0201 (12)	
H426	0.4401	0.0097	0.2326	0.024*	

### Atomic displacement parameters (Å^2^)

**Table:**

	*U*^11^	*U*^22^	*U*^33^	*U*^12^	*U*^13^	*U*^23^
Au1	0.01467 (11)	0.01186 (10)	0.01630 (11)	0.00198 (7)	0.00542 (8)	0.00180 (8)
S1	0.0256 (8)	0.0168 (7)	0.0209 (7)	−0.0008 (5)	0.0078 (6)	0.0022 (6)
P1	0.0143 (7)	0.0131 (6)	0.0138 (6)	0.0013 (5)	0.0046 (6)	0.0010 (5)
F1	0.039 (2)	0.031 (2)	0.046 (2)	0.0108 (17)	0.0080 (19)	0.0172 (18)
O1	0.049 (3)	0.020 (2)	0.031 (2)	−0.0057 (19)	0.019 (2)	−0.0058 (19)
N1	0.067 (6)	0.153 (9)	0.103 (7)	−0.006 (6)	0.023 (5)	0.088 (7)
C1	0.035 (4)	0.027 (3)	0.032 (4)	0.000 (3)	0.006 (3)	0.006 (3)
Au2	0.01510 (11)	0.01046 (10)	0.01807 (11)	−0.00068 (7)	0.00673 (8)	−0.00238 (8)
S2	0.0228 (8)	0.0291 (8)	0.0316 (8)	0.0023 (6)	0.0132 (7)	−0.0049 (7)
P2	0.0140 (7)	0.0121 (6)	0.0146 (6)	−0.0006 (5)	0.0050 (6)	−0.0010 (5)
F2	0.036 (2)	0.057 (3)	0.068 (3)	−0.012 (2)	0.008 (2)	0.023 (2)
O2	0.040 (3)	0.019 (2)	0.029 (2)	0.0025 (18)	0.008 (2)	0.0015 (18)
N2	0.032 (4)	0.064 (5)	0.055 (4)	0.000 (3)	0.004 (3)	−0.007 (4)
C2	0.021 (3)	0.032 (4)	0.029 (3)	0.000 (3)	0.005 (3)	−0.002 (3)
P3	0.0134 (7)	0.0118 (6)	0.0178 (7)	0.0015 (5)	0.0047 (6)	0.0009 (5)
F3	0.075 (3)	0.052 (3)	0.027 (2)	0.009 (2)	−0.006 (2)	−0.012 (2)
O3	0.031 (2)	0.034 (3)	0.041 (3)	0.0042 (19)	0.017 (2)	0.011 (2)
C3	0.052 (5)	0.041 (4)	0.055 (5)	−0.006 (3)	0.030 (4)	−0.005 (4)
P4	0.0146 (7)	0.0111 (6)	0.0192 (7)	−0.0010 (5)	0.0066 (6)	−0.0024 (5)
F4	0.027 (2)	0.077 (3)	0.027 (2)	−0.003 (2)	−0.0003 (17)	−0.001 (2)
O4	0.025 (2)	0.042 (3)	0.047 (3)	0.001 (2)	0.017 (2)	0.007 (2)
C4	0.035 (4)	0.065 (6)	0.058 (5)	0.004 (4)	0.011 (4)	0.026 (4)
F5	0.077 (3)	0.036 (2)	0.053 (3)	0.017 (2)	0.033 (3)	−0.004 (2)
O5	0.028 (3)	0.066 (4)	0.025 (2)	−0.003 (2)	0.005 (2)	−0.012 (2)
C5	0.087 (7)	0.045 (5)	0.054 (5)	−0.002 (4)	0.040 (5)	0.007 (4)
F6	0.026 (2)	0.070 (3)	0.040 (2)	−0.0067 (19)	0.0208 (18)	−0.004 (2)
O6	0.049 (3)	0.026 (2)	0.064 (3)	0.006 (2)	0.032 (3)	−0.006 (2)
C6	0.036 (4)	0.049 (5)	0.040 (4)	−0.010 (3)	0.015 (3)	−0.002 (4)
C11	0.013 (3)	0.016 (3)	0.014 (2)	0.002 (2)	0.002 (2)	0.002 (2)
C12	0.016 (3)	0.016 (3)	0.017 (3)	0.003 (2)	0.004 (2)	−0.005 (2)
C21	0.015 (3)	0.016 (3)	0.017 (3)	0.002 (2)	0.005 (2)	0.003 (2)
C22	0.021 (3)	0.010 (2)	0.023 (3)	−0.002 (2)	0.009 (2)	−0.001 (2)
C111	0.020 (3)	0.011 (2)	0.011 (2)	−0.002 (2)	0.005 (2)	0.001 (2)
C112	0.016 (3)	0.015 (3)	0.019 (3)	0.002 (2)	0.005 (2)	0.001 (2)
C113	0.022 (3)	0.028 (3)	0.014 (3)	0.001 (2)	0.001 (2)	−0.002 (2)
C114	0.032 (3)	0.022 (3)	0.020 (3)	−0.004 (2)	0.013 (3)	−0.004 (2)
C115	0.021 (3)	0.024 (3)	0.029 (3)	0.000 (2)	0.012 (3)	−0.002 (3)
C116	0.017 (3)	0.022 (3)	0.023 (3)	0.002 (2)	0.009 (2)	−0.001 (2)
C121	0.018 (3)	0.015 (3)	0.015 (3)	−0.002 (2)	0.007 (2)	−0.002 (2)
C122	0.016 (3)	0.034 (3)	0.023 (3)	−0.001 (2)	0.006 (2)	0.001 (3)
C123	0.023 (3)	0.053 (5)	0.026 (3)	−0.020 (3)	0.003 (3)	−0.004 (3)
C124	0.045 (4)	0.032 (4)	0.031 (3)	−0.021 (3)	0.022 (3)	−0.016 (3)
C125	0.047 (4)	0.017 (3)	0.033 (3)	0.000 (3)	0.024 (3)	−0.006 (3)
C126	0.023 (3)	0.019 (3)	0.021 (3)	0.002 (2)	0.009 (2)	0.001 (2)
C211	0.013 (3)	0.013 (2)	0.013 (2)	−0.0059 (19)	0.005 (2)	−0.006 (2)
C212	0.016 (3)	0.017 (3)	0.015 (3)	−0.002 (2)	0.001 (2)	−0.003 (2)
C213	0.023 (3)	0.025 (3)	0.021 (3)	−0.011 (2)	0.008 (3)	−0.004 (2)
C214	0.019 (3)	0.033 (3)	0.026 (3)	−0.013 (2)	0.015 (3)	−0.011 (3)
C215	0.017 (3)	0.024 (3)	0.029 (3)	−0.002 (2)	0.009 (3)	−0.012 (3)
C216	0.018 (3)	0.017 (3)	0.017 (3)	0.000 (2)	0.006 (2)	−0.003 (2)
C221	0.014 (3)	0.014 (3)	0.015 (2)	−0.002 (2)	0.004 (2)	−0.003 (2)
C222	0.018 (3)	0.016 (3)	0.017 (3)	0.000 (2)	0.005 (2)	−0.002 (2)
C223	0.018 (3)	0.026 (3)	0.020 (3)	0.006 (2)	0.003 (2)	0.001 (2)
C224	0.019 (3)	0.031 (3)	0.018 (3)	−0.002 (2)	0.006 (2)	−0.002 (2)
C225	0.022 (3)	0.023 (3)	0.021 (3)	−0.008 (2)	0.007 (3)	−0.007 (2)
C226	0.019 (3)	0.012 (3)	0.020 (3)	0.001 (2)	0.008 (2)	0.000 (2)
C311	0.010 (3)	0.018 (3)	0.020 (3)	0.005 (2)	0.002 (2)	−0.003 (2)
C312	0.022 (3)	0.017 (3)	0.032 (3)	0.000 (2)	0.011 (3)	−0.001 (2)
C313	0.022 (3)	0.026 (3)	0.023 (3)	0.005 (2)	0.005 (3)	0.002 (2)
C314	0.017 (3)	0.028 (3)	0.032 (3)	−0.004 (2)	0.002 (3)	−0.011 (3)
C315	0.028 (3)	0.026 (3)	0.042 (4)	−0.008 (3)	0.015 (3)	−0.001 (3)
C316	0.019 (3)	0.026 (3)	0.023 (3)	0.002 (2)	0.005 (2)	0.004 (2)
C321	0.014 (3)	0.018 (3)	0.019 (3)	0.004 (2)	0.003 (2)	0.000 (2)
C322	0.013 (3)	0.022 (3)	0.030 (3)	0.004 (2)	0.012 (2)	0.002 (2)
C323	0.018 (3)	0.022 (3)	0.049 (4)	0.000 (2)	0.008 (3)	−0.009 (3)
C324	0.023 (3)	0.042 (4)	0.029 (3)	−0.001 (3)	0.006 (3)	−0.020 (3)
C325	0.028 (3)	0.037 (4)	0.025 (3)	−0.004 (3)	0.013 (3)	−0.005 (3)
C326	0.022 (3)	0.021 (3)	0.026 (3)	−0.004 (2)	0.010 (3)	−0.003 (2)
C411	0.013 (3)	0.012 (3)	0.022 (3)	−0.002 (2)	0.003 (2)	0.001 (2)
C412	0.024 (3)	0.018 (3)	0.024 (3)	0.000 (2)	0.006 (3)	−0.005 (2)
C413	0.024 (3)	0.027 (3)	0.033 (3)	0.006 (2)	0.015 (3)	0.003 (3)
C414	0.018 (3)	0.025 (3)	0.036 (3)	−0.003 (2)	0.009 (3)	0.006 (3)
C415	0.020 (3)	0.023 (3)	0.028 (3)	−0.009 (2)	−0.001 (3)	−0.004 (3)
C416	0.028 (3)	0.019 (3)	0.026 (3)	−0.003 (2)	0.013 (3)	−0.004 (2)
C421	0.011 (3)	0.013 (2)	0.017 (3)	−0.0030 (19)	0.001 (2)	−0.004 (2)
C422	0.021 (3)	0.020 (3)	0.020 (3)	−0.001 (2)	0.003 (2)	−0.002 (2)
C423	0.035 (4)	0.025 (3)	0.019 (3)	−0.008 (3)	0.004 (3)	−0.003 (2)
C424	0.029 (3)	0.032 (3)	0.022 (3)	−0.015 (3)	0.009 (3)	−0.009 (3)
C425	0.022 (3)	0.026 (3)	0.029 (3)	−0.001 (2)	0.008 (3)	−0.008 (3)
C426	0.020 (3)	0.013 (3)	0.028 (3)	0.002 (2)	0.009 (2)	0.000 (2)

### Geometric parameters (Å, °)

**Table:**

Au1—P1	2.3079 (13)	C125—C126	1.373 (8)
Au1—P3	2.3105 (13)	C125—H125	0.9500
Au1—Au2	2.9220 (3)	C126—H126	0.9500
S1—O3	1.433 (4)	C211—C216	1.394 (7)
S1—O2	1.438 (4)	C211—C212	1.402 (7)
S1—O1	1.440 (4)	C212—C213	1.379 (7)
S1—C1	1.824 (6)	C212—H212	0.9500
P1—C121	1.797 (5)	C213—C214	1.379 (8)
P1—C111	1.804 (5)	C213—H213	0.9500
P1—C11	1.829 (5)	C214—C215	1.380 (8)
F1—C1	1.340 (7)	C214—H214	0.9500
N1—C4	1.121 (10)	C215—C216	1.390 (7)
C1—F3	1.323 (7)	C215—H215	0.9500
C1—F2	1.338 (7)	C216—H216	0.9500
Au2—P4	2.2993 (13)	C221—C226	1.390 (7)
Au2—P2	2.3052 (13)	C221—C222	1.407 (7)
S2—O6	1.430 (5)	C222—C223	1.390 (7)
S2—O5	1.437 (4)	C222—H222	0.9500
S2—O4	1.442 (4)	C223—C224	1.376 (8)
S2—C2	1.813 (6)	C223—H223	0.9500
P2—C221	1.811 (5)	C224—C225	1.390 (8)
P2—C211	1.812 (5)	C224—H224	0.9500
P2—C12	1.820 (5)	C225—C226	1.380 (7)
N2—C6	1.134 (9)	C225—H225	0.9500
C2—F6	1.322 (7)	C226—H226	0.9500
C2—F4	1.326 (6)	C311—C312	1.382 (7)
C2—F5	1.343 (7)	C311—C316	1.389 (7)
P3—C321	1.812 (5)	C312—C313	1.382 (8)
P3—C311	1.819 (5)	C312—H312	0.9500
P3—C21	1.823 (5)	C313—C314	1.371 (8)
C3—C4	1.430 (10)	C313—H313	0.9500
C3—H3A	0.9800	C314—C315	1.408 (8)
C3—H3B	0.9800	C314—H314	0.9500
C3—H3C	0.9800	C315—C316	1.380 (8)
P4—C421	1.807 (5)	C315—H315	0.9500
P4—C411	1.816 (5)	C316—H316	0.9500
P4—C22	1.826 (5)	C321—C326	1.384 (7)
C5—C6	1.437 (10)	C321—C322	1.392 (7)
C5—H5A	0.9800	C322—C323	1.380 (8)
C5—H5B	0.9800	C322—H322	0.9500
C5—H5C	0.9800	C323—C324	1.371 (9)
C11—C12	1.525 (7)	C323—H323	0.9500
C11—H11A	0.9900	C324—C325	1.383 (9)
C11—H11B	0.9900	C324—H324	0.9500
C12—H12A	0.9900	C325—C326	1.385 (8)
C12—H12B	0.9900	C325—H325	0.9500
C21—C22	1.536 (7)	C326—H326	0.9500
C21—H21A	0.9900	C411—C412	1.382 (7)
C21—H21B	0.9900	C411—C416	1.383 (7)
C22—H22A	0.9900	C412—C413	1.382 (8)
C22—H22B	0.9900	C412—H412	0.9500
C111—C112	1.394 (7)	C413—C414	1.385 (8)
C111—C116	1.398 (7)	C413—H413	0.9500
C112—C113	1.387 (7)	C414—C415	1.375 (8)
C112—H112	0.9500	C414—H414	0.9500
C113—C114	1.393 (8)	C415—C416	1.395 (8)
C113—H113	0.9500	C415—H415	0.9500
C114—C115	1.377 (7)	C416—H416	0.9500
C114—H114	0.9500	C421—C422	1.385 (7)
C115—C116	1.373 (7)	C421—C426	1.409 (7)
C115—H115	0.9500	C422—C423	1.397 (7)
C116—H116	0.9500	C422—H422	0.9500
C121—C122	1.381 (7)	C423—C424	1.373 (8)
C121—C126	1.403 (7)	C423—H423	0.9500
C122—C123	1.398 (8)	C424—C425	1.368 (8)
C122—H122	0.9500	C424—H424	0.9500
C123—C124	1.367 (9)	C425—C426	1.377 (7)
C123—H123	0.9500	C425—H425	0.9500
C124—C125	1.377 (9)	C426—H426	0.9500
C124—H124	0.9500		
			
P1—Au1—P3	171.77 (5)	C122—C123—H123	119.8
P1—Au1—Au2	92.77 (3)	C123—C124—C125	120.6 (6)
P3—Au1—Au2	95.17 (3)	C123—C124—H124	119.7
O3—S1—O2	116.0 (3)	C125—C124—H124	119.7
O3—S1—O1	114.9 (3)	C126—C125—C124	119.8 (6)
O2—S1—O1	114.3 (2)	C126—C125—H125	120.1
O3—S1—C1	103.7 (3)	C124—C125—H125	120.1
O2—S1—C1	102.8 (3)	C125—C126—C121	120.3 (5)
O1—S1—C1	102.6 (3)	C125—C126—H126	119.9
C121—P1—C111	106.7 (2)	C121—C126—H126	119.9
C121—P1—C11	106.2 (2)	C216—C211—C212	119.2 (5)
C111—P1—C11	105.1 (2)	C216—C211—P2	123.6 (4)
C121—P1—Au1	114.46 (18)	C212—C211—P2	117.1 (4)
C111—P1—Au1	107.50 (17)	C213—C212—C211	120.3 (5)
C11—P1—Au1	116.15 (17)	C213—C212—H212	119.9
F3—C1—F2	108.1 (5)	C211—C212—H212	119.9
F3—C1—F1	107.4 (5)	C214—C213—C212	120.1 (5)
F2—C1—F1	106.9 (5)	C214—C213—H213	120.0
F3—C1—S1	112.0 (5)	C212—C213—H213	120.0
F2—C1—S1	110.4 (4)	C213—C214—C215	120.4 (5)
F1—C1—S1	111.9 (4)	C213—C214—H214	119.8
P4—Au2—P2	177.10 (5)	C215—C214—H214	119.8
P4—Au2—Au1	88.13 (3)	C214—C215—C216	120.3 (5)
P2—Au2—Au1	89.72 (3)	C214—C215—H215	119.8
O6—S2—O5	117.2 (3)	C216—C215—H215	119.8
O6—S2—O4	113.8 (3)	C215—C216—C211	119.7 (5)
O5—S2—O4	114.3 (3)	C215—C216—H216	120.1
O6—S2—C2	102.5 (3)	C211—C216—H216	120.1
O5—S2—C2	102.9 (3)	C226—C221—C222	119.1 (5)
O4—S2—C2	103.5 (3)	C226—C221—P2	121.8 (4)
C221—P2—C211	106.8 (2)	C222—C221—P2	119.1 (4)
C221—P2—C12	106.9 (2)	C223—C222—C221	119.9 (5)
C211—P2—C12	106.3 (2)	C223—C222—H222	120.0
C221—P2—Au2	113.02 (17)	C221—C222—H222	120.0
C211—P2—Au2	112.79 (16)	C224—C223—C222	120.0 (5)
C12—P2—Au2	110.66 (17)	C224—C223—H223	120.0
F6—C2—F4	107.9 (5)	C222—C223—H223	120.0
F6—C2—F5	106.8 (5)	C223—C224—C225	120.4 (5)
F4—C2—F5	106.7 (5)	C223—C224—H224	119.8
F6—C2—S2	111.9 (4)	C225—C224—H224	119.8
F4—C2—S2	112.1 (4)	C226—C225—C224	120.0 (5)
F5—C2—S2	111.2 (4)	C226—C225—H225	120.0
C321—P3—C311	108.1 (2)	C224—C225—H225	120.0
C321—P3—C21	107.7 (2)	C225—C226—C221	120.5 (5)
C311—P3—C21	104.3 (2)	C225—C226—H226	119.7
C321—P3—Au1	111.19 (17)	C221—C226—H226	119.7
C311—P3—Au1	108.64 (17)	C312—C311—C316	119.2 (5)
C21—P3—Au1	116.49 (17)	C312—C311—P3	123.2 (4)
C4—C3—H3A	109.5	C316—C311—P3	117.5 (4)
C4—C3—H3B	109.5	C313—C312—C311	120.2 (5)
H3A—C3—H3B	109.5	C313—C312—H312	119.9
C4—C3—H3C	109.5	C311—C312—H312	119.9
H3A—C3—H3C	109.5	C314—C313—C312	121.2 (5)
H3B—C3—H3C	109.5	C314—C313—H313	119.4
C421—P4—C411	109.0 (2)	C312—C313—H313	119.4
C421—P4—C22	107.4 (2)	C313—C314—C315	119.0 (5)
C411—P4—C22	102.0 (2)	C313—C314—H314	120.5
C421—P4—Au2	113.18 (17)	C315—C314—H314	120.5
C411—P4—Au2	112.60 (18)	C316—C315—C314	119.6 (6)
C22—P4—Au2	111.96 (17)	C316—C315—H315	120.2
N1—C4—C3	176.0 (11)	C314—C315—H315	120.2
C6—C5—H5A	109.5	C315—C316—C311	120.7 (5)
C6—C5—H5B	109.5	C315—C316—H316	119.6
H5A—C5—H5B	109.5	C311—C316—H316	119.6
C6—C5—H5C	109.5	C326—C321—C322	119.7 (5)
H5A—C5—H5C	109.5	C326—C321—P3	118.6 (4)
H5B—C5—H5C	109.5	C322—C321—P3	121.6 (4)
N2—C6—C5	178.7 (9)	C323—C322—C321	120.3 (5)
C12—C11—P1	118.0 (3)	C323—C322—H322	119.8
C12—C11—H11A	107.8	C321—C322—H322	119.8
P1—C11—H11A	107.8	C324—C323—C322	119.6 (6)
C12—C11—H11B	107.8	C324—C323—H323	120.2
P1—C11—H11B	107.8	C322—C323—H323	120.2
H11A—C11—H11B	107.1	C323—C324—C325	120.8 (6)
C11—C12—P2	116.0 (3)	C323—C324—H324	119.6
C11—C12—H12A	108.3	C325—C324—H324	119.6
P2—C12—H12A	108.3	C324—C325—C326	119.9 (6)
C11—C12—H12B	108.3	C324—C325—H325	120.0
P2—C12—H12B	108.3	C326—C325—H325	120.0
H12A—C12—H12B	107.4	C321—C326—C325	119.6 (5)
C22—C21—P3	119.2 (4)	C321—C326—H326	120.2
C22—C21—H21A	107.5	C325—C326—H326	120.2
P3—C21—H21A	107.5	C412—C411—C416	119.9 (5)
C22—C21—H21B	107.5	C412—C411—P4	117.0 (4)
P3—C21—H21B	107.5	C416—C411—P4	123.1 (4)
H21A—C21—H21B	107.0	C411—C412—C413	120.3 (5)
C21—C22—P4	117.9 (3)	C411—C412—H412	119.8
C21—C22—H22A	107.8	C413—C412—H412	119.8
P4—C22—H22A	107.8	C412—C413—C414	119.7 (5)
C21—C22—H22B	107.8	C412—C413—H413	120.1
P4—C22—H22B	107.8	C414—C413—H413	120.1
H22A—C22—H22B	107.2	C415—C414—C413	120.4 (5)
C112—C111—C116	118.4 (5)	C415—C414—H414	119.8
C112—C111—P1	123.5 (4)	C413—C414—H414	119.8
C116—C111—P1	118.0 (4)	C414—C415—C416	119.8 (5)
C113—C112—C111	120.5 (5)	C414—C415—H415	120.1
C113—C112—H112	119.8	C416—C415—H415	120.1
C111—C112—H112	119.8	C411—C416—C415	119.9 (5)
C112—C113—C114	119.8 (5)	C411—C416—H416	120.1
C112—C113—H113	120.1	C415—C416—H416	120.1
C114—C113—H113	120.1	C422—C421—C426	118.9 (5)
C115—C114—C113	120.1 (5)	C422—C421—P4	119.2 (4)
C115—C114—H114	119.9	C426—C421—P4	121.9 (4)
C113—C114—H114	119.9	C421—C422—C423	120.1 (5)
C116—C115—C114	120.0 (5)	C421—C422—H422	120.0
C116—C115—H115	120.0	C423—C422—H422	120.0
C114—C115—H115	120.0	C424—C423—C422	120.0 (6)
C115—C116—C111	121.1 (5)	C424—C423—H423	120.0
C115—C116—H116	119.4	C422—C423—H423	120.0
C111—C116—H116	119.4	C425—C424—C423	120.3 (6)
C122—C121—C126	119.5 (5)	C425—C424—H424	119.8
C122—C121—P1	120.5 (4)	C423—C424—H424	119.8
C126—C121—P1	120.0 (4)	C424—C425—C426	120.8 (6)
C121—C122—C123	119.3 (6)	C424—C425—H425	119.6
C121—C122—H122	120.3	C426—C425—H425	119.6
C123—C122—H122	120.3	C425—C426—C421	119.9 (5)
C124—C123—C122	120.4 (6)	C425—C426—H426	120.1
C124—C123—H123	119.8	C421—C426—H426	120.1
			
Au2—Au1—P1—C121	118.83 (19)	C12—P2—C211—C212	171.0 (4)
Au2—Au1—P1—C111	−122.84 (18)	Au2—P2—C211—C212	49.6 (4)
Au2—Au1—P1—C11	−5.49 (18)	C216—C211—C212—C213	−2.3 (7)
O3—S1—C1—F3	−58.3 (5)	P2—C211—C212—C213	−179.2 (4)
O2—S1—C1—F3	62.9 (5)	C211—C212—C213—C214	2.0 (8)
O1—S1—C1—F3	−178.2 (4)	C212—C213—C214—C215	−1.4 (8)
O3—S1—C1—F2	−178.7 (4)	C213—C214—C215—C216	1.0 (8)
O2—S1—C1—F2	−57.6 (5)	C214—C215—C216—C211	−1.3 (8)
O1—S1—C1—F2	61.4 (5)	C212—C211—C216—C215	1.9 (7)
O3—S1—C1—F1	62.3 (5)	P2—C211—C216—C215	178.6 (4)
O2—S1—C1—F1	−176.5 (4)	C211—P2—C221—C226	−70.3 (5)
O1—S1—C1—F1	−57.6 (5)	C12—P2—C221—C226	43.1 (5)
P1—Au1—Au2—P4	130.95 (5)	Au2—P2—C221—C226	165.1 (4)
P3—Au1—Au2—P4	−46.89 (5)	C211—P2—C221—C222	109.6 (4)
P1—Au1—Au2—P2	−47.11 (5)	C12—P2—C221—C222	−136.9 (4)
P3—Au1—Au2—P2	135.05 (5)	Au2—P2—C221—C222	−15.0 (5)
Au1—Au2—P2—C221	−46.58 (18)	C226—C221—C222—C223	−1.1 (8)
Au1—Au2—P2—C211	−167.81 (18)	P2—C221—C222—C223	179.0 (4)
Au1—Au2—P2—C12	73.24 (19)	C221—C222—C223—C224	0.3 (8)
O6—S2—C2—F6	−60.5 (5)	C222—C223—C224—C225	0.2 (8)
O5—S2—C2—F6	61.5 (5)	C223—C224—C225—C226	0.1 (8)
O4—S2—C2—F6	−179.1 (4)	C224—C225—C226—C221	−0.8 (8)
O6—S2—C2—F4	60.9 (5)	C222—C221—C226—C225	1.4 (8)
O5—S2—C2—F4	−177.0 (4)	P2—C221—C226—C225	−178.7 (4)
O4—S2—C2—F4	−57.7 (5)	C321—P3—C311—C312	−102.9 (5)
O6—S2—C2—F5	−179.8 (4)	C21—P3—C311—C312	11.5 (5)
O5—S2—C2—F5	−57.7 (5)	Au1—P3—C311—C312	136.3 (4)
O4—S2—C2—F5	61.6 (5)	C321—P3—C311—C316	77.4 (5)
Au2—Au1—P3—C321	119.99 (19)	C21—P3—C311—C316	−168.2 (4)
Au2—Au1—P3—C311	−121.21 (18)	Au1—P3—C311—C316	−43.4 (5)
Au2—Au1—P3—C21	−3.91 (19)	C316—C311—C312—C313	−2.1 (8)
Au1—Au2—P4—C421	−48.35 (18)	P3—C311—C312—C313	178.2 (4)
Au1—Au2—P4—C411	−172.49 (19)	C311—C312—C313—C314	0.8 (8)
Au1—Au2—P4—C22	73.23 (19)	C312—C313—C314—C315	0.6 (9)
C121—P1—C11—C12	−55.4 (4)	C313—C314—C315—C316	−0.6 (9)
C111—P1—C11—C12	−168.2 (4)	C314—C315—C316—C311	−0.7 (9)
Au1—P1—C11—C12	73.1 (4)	C312—C311—C316—C315	2.1 (8)
P1—C11—C12—P2	−56.9 (5)	P3—C311—C316—C315	−178.2 (4)
C221—P2—C12—C11	94.4 (4)	C311—P3—C321—C326	−89.9 (5)
C211—P2—C12—C11	−151.8 (4)	C21—P3—C321—C326	158.0 (4)
Au2—P2—C12—C11	−29.0 (4)	Au1—P3—C321—C326	29.3 (5)
C321—P3—C21—C22	−58.8 (4)	C311—P3—C321—C322	91.9 (5)
C311—P3—C21—C22	−173.5 (4)	C21—P3—C321—C322	−20.2 (5)
Au1—P3—C21—C22	66.8 (4)	Au1—P3—C321—C322	−149.0 (4)
P3—C21—C22—P4	−51.2 (5)	C326—C321—C322—C323	−0.1 (8)
C421—P4—C22—C21	91.8 (4)	P3—C321—C322—C323	178.2 (4)
C411—P4—C22—C21	−153.6 (4)	C321—C322—C323—C324	1.5 (8)
Au2—P4—C22—C21	−33.0 (4)	C322—C323—C324—C325	−1.5 (9)
C121—P1—C111—C112	−112.8 (5)	C323—C324—C325—C326	0.0 (9)
C11—P1—C111—C112	−0.3 (5)	C322—C321—C326—C325	−1.4 (8)
Au1—P1—C111—C112	124.0 (4)	P3—C321—C326—C325	−179.7 (4)
C121—P1—C111—C116	69.1 (5)	C324—C325—C326—C321	1.4 (9)
C11—P1—C111—C116	−178.5 (4)	C421—P4—C411—C412	−163.5 (4)
Au1—P1—C111—C116	−54.2 (4)	C22—P4—C411—C412	83.2 (5)
C116—C111—C112—C113	−0.3 (8)	Au2—P4—C411—C412	−37.0 (5)
P1—C111—C112—C113	−178.4 (4)	C421—P4—C411—C416	18.7 (5)
C111—C112—C113—C114	1.3 (8)	C22—P4—C411—C416	−94.7 (5)
C112—C113—C114—C115	−2.6 (9)	Au2—P4—C411—C416	145.1 (4)
C113—C114—C115—C116	2.8 (9)	C416—C411—C412—C413	0.3 (8)
C114—C115—C116—C111	−1.8 (8)	P4—C411—C412—C413	−177.6 (4)
C112—C111—C116—C115	0.5 (8)	C411—C412—C413—C414	−0.8 (9)
P1—C111—C116—C115	178.7 (4)	C412—C413—C414—C415	1.0 (9)
C111—P1—C121—C122	−106.2 (4)	C413—C414—C415—C416	−0.6 (9)
C11—P1—C121—C122	142.1 (4)	C412—C411—C416—C415	0.0 (8)
Au1—P1—C121—C122	12.6 (5)	P4—C411—C416—C415	177.8 (4)
C111—P1—C121—C126	74.7 (5)	C414—C415—C416—C411	0.1 (9)
C11—P1—C121—C126	−37.1 (5)	C411—P4—C421—C422	107.3 (4)
Au1—P1—C121—C126	−166.5 (4)	C22—P4—C421—C422	−142.9 (4)
C126—C121—C122—C123	0.8 (8)	Au2—P4—C421—C422	−18.8 (5)
P1—C121—C122—C123	−178.3 (4)	C411—P4—C421—C426	−75.3 (5)
C121—C122—C123—C124	−0.2 (9)	C22—P4—C421—C426	34.5 (5)
C122—C123—C124—C125	−0.4 (9)	Au2—P4—C421—C426	158.6 (4)
C123—C124—C125—C126	0.3 (9)	C426—C421—C422—C423	−0.3 (8)
C124—C125—C126—C121	0.3 (8)	P4—C421—C422—C423	177.1 (4)
C122—C121—C126—C125	−0.9 (8)	C421—C422—C423—C424	−1.4 (8)
P1—C121—C126—C125	178.3 (4)	C422—C423—C424—C425	2.8 (9)
C221—P2—C211—C216	108.1 (4)	C423—C424—C425—C426	−2.5 (9)
C12—P2—C211—C216	−5.8 (5)	C424—C425—C426—C421	0.7 (8)
Au2—P2—C211—C216	−127.2 (4)	C422—C421—C426—C425	0.7 (8)
C221—P2—C211—C212	−75.2 (4)	P4—C421—C426—C425	−176.7 (4)

### Hydrogen-bond geometry (Å, °)

**Table:**

*D*—H···*A*	*D*—H	H···*A*	*D*···*A*	*D*—H···*A*
C212—H212···O1^i^	0.95	2.45	3.387 (7)	171
C21—H21B···O1^i^	0.99	2.34	3.268 (7)	155
C11—H11B···O4^ii^	0.99	2.36	3.301 (7)	158

Symmetry codes: (i) *x*, −*y*+1/2, *z*+1/2; (ii) *x*, −*y*+1/2, *z*−1/2.
